# Evolutionary History of the Glycoside Hydrolase 3 (GH3) Family Based on the Sequenced Genomes of 48 Plants and Identification of Jasmonic Acid-Related GH3 Proteins in *Solanum tuberosum*

**DOI:** 10.3390/ijms19071850

**Published:** 2018-06-23

**Authors:** Chao Zhang, Leilei Zhang, Dongdong Wang, Haoli Ma, Bailin Liu, Zheng Shi, Xiaohui Ma, Yue Chen, Qin Chen

**Affiliations:** 1 State Key Laboratory of Crop Stress Biology for Arid Areas, College of Agronomy, Northwest A&F University, Yangling 712100, Shaanxi, China; zhangchao520@nwafu.edu.cn (C.Z.); dongdong-1025@hotmail.com (D.W.); mahaoli@nwsuaf.edu.cn (H.M.); liubl@nwafu.edu.cn (B.L.); summer_shi@nwafu.edu.cn (Z.S.); maxiaohui1216@nwafu.edu.cn (X.M.); 2 College of Agronomy, Liaocheng University, Liaocheng 252059, Shandong, China; zhangleilei@lcu.edu.cn

**Keywords:** GH3 family, sequencing plants, potato, jasmonic acids, tissues, biotic

## Abstract

Glycoside Hydrolase 3 (GH3) is a phytohormone-responsive family of proteins found in many plant species. These proteins contribute to the biological activity of indolacetic acid (IAA), jasmonic acid (JA), and salicylic acid (SA). They also affect plant growth and developmental processes as well as some types of stress. In this study, *GH3* genes were identified in 48 plant species, including algae, mosses, ferns, gymnosperms, and angiosperms. No GH3 representative protein was found in algae, but we identified 4 genes in mosses, 19 in ferns, 7 in gymnosperms, and several in angiosperms. The results showed that GH3 proteins are mainly present in seed plants. Phylogenetic analysis of all GH3 proteins showed three separate clades. Group I was related to JA adenylation, group II was related to IAA adenylation, and group III was separated from group II, but its function was not clear. The structure of the GH3 proteins indicated highly conserved sequences in the plant kingdom. The analysis of JA adenylation in relation to gene expression of GH3 in potato (*Solanum tuberosum*) showed that *StGH3.12* greatly responded to methyl jasmonate (MeJA) treatment. The expression levels of *StGH3.1*, *StGH3.11*, and *StGH3.12* were higher in the potato flowers, and *StGH3.11* expression was also higher in the stolon. Our research revealed the evolution of the GH3 family, which is useful for studying the precise function of GH3 proteins related to JA adenylation in *S. tuberosum* when the plants are developing and under biotic stress.

## 1. Introduction

Glycoside Hydrolase 3 (GH3) proteins are widespread in plants. In Hagen et al. research, differential hybridization screening was used to first isolate GH3 proteins in an auxin-induced cDNA clone from etiolated soybean hypocotyls [1]. Afterward, GH3 proteins were identified from *Nicotiana tabacum* L. [2], *Arabidopsis* [3], *Oryza sativa* [4], and other plants [5,6,7,8]. The GH3 proteins were identified in mosses, *Physcomitrella patens* [9], and *Marchantia polymorpha* L. [10], which were present on earth earlier than land plants by nearly 400 million years. The analysis of promotor sequences showed that some plant hormone elements exist in GH3 promotor sequences [11]. Subsequent research proved that these proteins adjust to phytohormone signaling pathways. A two-step mechanism showed that the GH3 family conjugates amino acids to form diverse acyl acid substrates. The first step indicates adenylation of an acyl acid hormone and the release of pyrophosphate. In the second transfer step, the amine group of an amino acid nucleophilically displaces AMP, which creates the conjugated acyl acid [12]. Only the *AtGH3.11* (*JAR1*) response requires ATP. The rest of the GH3 proteins can be developed in the absence of ATP [13]. The GH3 proteins regulate the levels of phytohormones, including SA, JA, and IAA [14]. Group I GH3 proteins catalyze the conjugation of additional amino acids to form jasmonoyl-isoleucine (JA-Ile), and Group II GH3 proteins catalyze the conjugation of additional amino acids to form IAA complexes. Jagadeeswaran et al. inferred that *Arabidopsis* GH3.12 (*PBS3*) may work upstream of SA [15]. In published phylogenetic trees of *Arabidopsis*, the GH3 proteins are divided into three major groups on the basis of sequence similarity and acyl substrate specificity [16]. Group I proteins use JA as a substrate, which promotes the formation of JA-Ile and boosts JA-mediated responses [8]. JA has been recognized as vital for defense. It is synthesized within minutes to regulate the expression of many genes and affects the resistance to herbivory and disease through the JA biological pathway [17]. The research of Havko et al. showed that JA regulates plant growth and defense through JAZ-DELLA interaction, which influences the ability of DELLAs to repress phytochrome-interacting factor (PIF) transcription factors in plant growth. JAZ-DELLA interaction could also interfere with MYC transcription factors in plant defenses. However, the photoreceptor phytochrome B (phyB) is able to prevent PIFs expression, which influences growth and JA effects on photosynthesis. Therefore, JA plays an important role in growth-defense mechanisms [18]. However, Compos et al. pointed out that the balance between growth and defense is orchestrated by a hormone-based transcriptional network that is hardwired to restrict growth when activating the JA pathway but was not activated by the diversion of photo-assimilates from growth to defense [19]. *AtGH3.11*/*JAR1* was identified as a pre-receptor in jasmonate responses [14]. At the same time, a *JAR1* mutant was involved in the resistance triggered by non-pathogenic bacteria and was able to reduce the damage of ozone to *Arabidopsis* [20,21]. Heitz et al. pointed out that the JA-Ile derivatives 12OH-JA-Ile and 12COOH-JA-Ile accumulated in injured *Arabidopsis* leaves and were regulated by Cytochromes P450, CYP94C1, and CYP94B3. The elucidation of CYP94-mediated JA-Ile oxidation opens new avenues for understanding JA metabolism and signaling [22].

Group II proteins can conjugate IAA to the free IAA and store phytohormones in an inactive form [23]. *AtGH3.12/PBS3* belongs to Group III and uses 4-hydroxybenzoate (4HBA) with a higher efficiency than SA [24]. Until recently, the functions of GH3 proteins had been reported primarily in *Arabidopsis*. *AtGH3.2*/*Ydk1-D* can promote short primary roots and reduce lateral root numbers [25]. Additionally, *AtGH3.6/dfl1* restrains shoot elongation and lateral root formation [26], and *AtGH3.9* seems to be specifically active in roots [27].

The phytohormone IAA is responsible for growth and development in flowering plants. It influences cell division, expansion, and differentiation [28,29]. Group II genes of the GH3 family are involved in IAA regulation. Similarly, JA is one of the major plant hormones involved in regulating growth and biotic defense [30]. Many research studies state that JA participates in biotic stresses in plants. Therefore, group I genes not only take part in growth but also have key roles in biotic stress on the basis of JA biological function in plants.

With the continuous improvement of gene sequencing technology, genetic data have been generated for more species. The GH3 family could be identified in several plants, but no researchers have described a detailed and complete evolutionary history. Terol et al. displayed the GH3 evolutionary history in 20 plant species through EST (Expressed Sequence Tag) analysis [31]. Sequences of complete genomes were found for at least 48 plants which covered stages from lower to higher plants. Therefore, the evolution of GH3 proteins may be studied more accurately on the basis of these sequenced plant species. We also analyzed the GH3 family in 48 species, which included algae, mosses, ferns, gymnosperms, and angiosperms. We used bioinformatics methods. Furthermore, we analyzed the gene characteristics, structures, phylogenetic relationships, gene ontology (GO) annotations, and expression patterns of the GH3 proteins in *Solanum tuberosum*. Therefore, the results of this study not only reveal the evolution of the GH3 family but also provide some useful information for further research on the potato.

## 2. Results and Discussion

### 2.1. The GH3 Family Identified in 48 Plant Species

To study the evolution of the GH3 family, we surveyed the sequences of GH3 family members in sequenced plant species. We used the GH3 full-length protein sequence of *Arabidopsis thaliana* [16] as a query to search the available genome sequences from 48 plant species including algae, mosses, ferns, gymnosperms, and angiosperms. We found a total of 579 proteins in 48 plants. The number of GH3 proteins ranged from 0 to 38 (Table 1). According to the evolutionary relationships in plants, no GH3 proteins were acquired in algae. Only two proteins were identified in each moss known as *Marchantia polymorpha* L. and *Physcomitrella patens*. The fern species *Selaginella moellendorffii* contained 19 GH3 proteins, which was a large group compared with some other plants. We could not find genomic information for the gymnosperm *Picea abies* in the NCBI Genome, but it was reported by Nystedt et al. [32], and seven GH3 proteins were identified in it. Numerous angiosperms have been sequenced, which makes it easier to determine how many GH3 genes they have acquired. The highest number of GH3 proteins was found in *Brassica rapa* but it does not have the largest genome. Different plants had different GH3 proteins, but these numbers were not correlated with the genome length. According to the general trend, the GH3 protein appeared first in mosses and, as plants continued to evolve, more GH3 genes were found. We found that the number of GH3 family proteins increased with the emergence of seed plants (ferns, gymnosperms, and angiosperms).

By analyzing the number of each GH3 protein group among the 48 plants, we found that group II was largely present, while group III existed only in a few plant species. Although there were two proteins belonging to group I in *P. patens*, Stumpe et al. indicated that they do not have the function of *JAR1*, which means they do not take part in the JA biological pathway [33]. Similarly, Bierfreund et al. showed that the defense against Botrytis infection involved SA and 12-oxo-phytodienoic acid, which promote the reinforcement of the cell wall, programmed cell death, and accumulation of evolutionary conserved defense signals. JA did not play any role in these processes [9]. Bowman et al. indicated that *M. polymorpha* could synthesize the JA precursor 12-oxo-phytodienoic acid (OPDA) but it lacks the OPDA reductase (OPR3) that produces the vascular plant hormone JA-Ile. The GH3 protein involved belongs to group I, which had no function [10,34]. In summary, mosses contained GH3 proteins and some of these proteins belonged to group I, but they did not play roles in the JA biological pathway. Therefore, the classification of the GH3 family through protein sequence comparison only served as a reference. Gene detail function in each plant, especially in ancient plant species, needed to be verified by gene function analysis.

### 2.2. Analysis of the Phylogenetic Tree and Duplication Patterns

**Figure 1:**
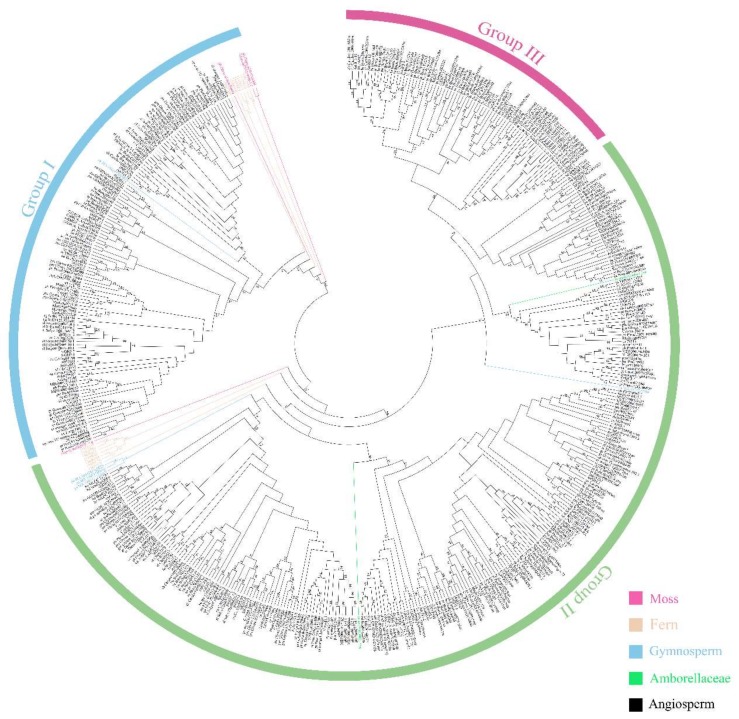
Unrooted Neighbor-Joining tree constructs from GH3 proteins of 48 plant species. The protein names and lines having different colors in the phylogenetic tree represent the proteins belonging to different lineages. Information for each color is displayed in the lower right corner of the figure. Subfamilies are distinguished by three curves with different colors. **Blue**, **green**, and **purple** indicate group I, II, and III, respectively.

The phylogenetic tree of the GH3 members in 48 plant species was constructed on the basis of the similarities of the protein sequences (Figure 1 and Table S1). The tree contained three clades, which is consistent with previous studies [4,5,38]. The functional groups known as group I and group II, which are related to JA and IAA, respectively, were present in all major plant lineages, including the mosses. Three genes from mosses could be assigned to group I, which contained the most ancient genes. The GH3 proteins may have played important roles in environmental adaptation in mosses. This gene group was present in all seed plants. It could be inferred that the function of group I was indispensable to all plants. One GH3 protein belonged to group II in mosses, but more group II proteins appeared in seed plants. This suggests that group II had a key role in plant evolution. Group III proteins were only present in eudicots. From the phylogenetic tree analysis, group III was closer to group II and belonged to one of the branches. Moreover, group II and III had the same plant lineages and evolved from the moss *M. polymorpha* L. (*Mapoly0053s0073.1.p*). Group III members were found in eudicots, including all Brassicaceaes (*A. thaliana*, *Brassica oleracea*, *B. rapa*, *Capsella rubella*, and *Arabidopsis lyrata*), *Gossypium raimondii*, *Brachypodium distachyon*, and *Theobroma cacao*. This result is consistent with those of Singh et al. [39]. Therefore, we speculated that group III contains the most recent evolutionary members. Okrent et al. suggested that group III proteins play a role in the response to biotic and abiotic stress [14].

Group I and II GH3 proteins have the same ancient origin, and the evolutionary explosion of these groups was caused by many duplication events. Early duplication events were identified in group I and II of ferns (Figure 1).

For a further understanding of the GH3 evolutionary relationships, the prediction of the molecular evolution rates could clarify the gene evolution process. The selective pressure was estimated by calculating the ratio of the nonsynonymous substitution rate to the synonymous substitution rate (Ka/Ks value), which allowed us to analyze the molecular evolutionary rates (Table S2). Ka/Ks values were calculated for each duplication event and showed that almost every gene pair evolved at a Ka/Ks value lower than one, except the *ta__Thecc1EG031553*/*ta__Thecc1EG031554* gene pair in *T. cacao* (the Ka/Ks value is 1.638). This gene pair experienced a positive selection pressure in evolution. The other gene pairs indicated that the gene was subject to purification selection. Compared with each stage duplication event, the angiosperms showed extensive duplication. We first determined the duplication event in ferns. That Ka/Ks Values were <1, and two kinds of duplication features had similar values (0.318 to 0.388) in *S. moellendorffii*. The number of GH3 duplications in dicots was higher than in monocots. Plenty of tandem and segmental duplications occurred in *Brassicaceae*. The most segmental duplications were identified in *Glycine max*, and there were no tandem duplications. The number of GH3 proteins and duplication events suggested that the increase in GH3 proteins was due to numerous duplication events in angiosperms. Plants evolve with changes in the environment, and positive selection promoted the exchange of gene functions to survive. Therefore, the selective patterns can partly explain the evolutionary patterns of the genes.

### 2.3. Structural Analysis of GH3 Proteins

**Figure 2:**
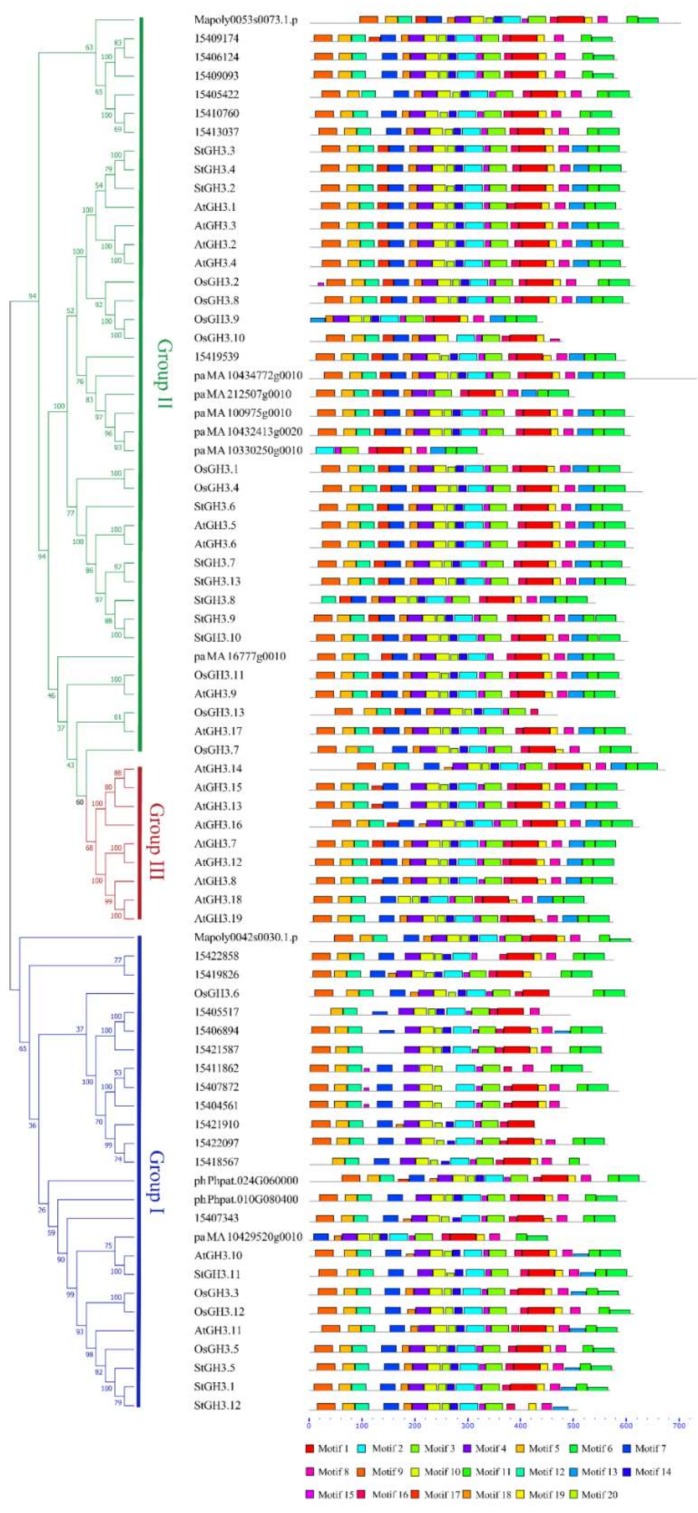
Gene motifs of seven plant species. The species belong to different lineages, including *M.a polymorpha* L., *P. patens*, *S. moellendorffii*, *P. abies*, *S. tuberosum*, *A. thaliana* and *O. sativa*. On the left, the protein name and phylogenetic tree are shown. The different color boxes indicate 20 motifs, which were found by using the MEME program.

Seven species from different stages, from moss to angiosperm, were selected as the candidates to analyze their structures. The prediction of protein motifs was an essential method for protein analysis. The motifs of GH3 proteins were identified using the MEME website. Twenty motifs were analyzed in these proteins (Figure 2). The GH3 protein family members have highly conserved motifs. The vast majority of proteins contained about 20 motifs and they were in the same order. The moss and fern species had 17–19 motifs. Motif 17 did not exist and was analyzed by the MEME program. The PaGH3 protein (pa__MA_10330250g0010) only had 10 motifs, which was the lowest number of motifs in all proteins. Correspondingly, it also had the shortest length. In comparison to other gene families such as the WRKY family [40], the motifs of subgroup proteins showed no significant differences.

**Figure 3:**
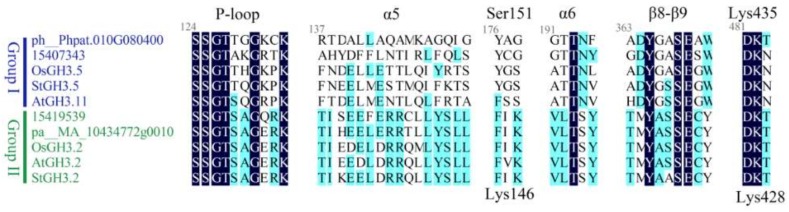
Predictions of protein structure by analyzing 10 GH3 proteins. The selected proteins belonged to groups I and II. AtGH3.11 plays a key role in the JA pathway, and its mutant is named JAR1 [13]. YDK1-D is an AtGH3.2 mutant and responds to auxin [25]. The **grey** numbers (124, 137, 176, and so on) stand for the location of amino acids. The **black** annotates symbolize the secondary structure of the protein.

The GH3 family conjugates amino acids to diverse acyl acid substrates. We analyzed the protein sequences, which showed that the auxin-responsive and JA-responsive proteins had different structures. The prediction of protein secondary structures showed different main structures in each group. We took 10 proteins as candidates to analyze the information from Westfall et al. [8]. These 10 proteins belonged to four different evolutionary stages and contained α5 and α6, which provide a hydrophobic pocket for the pentenyl tail, β8-9, and P-loop. These were conserved structures in each protein (Figure 3). The alignment of proteins from each group showed highly conserved lysine residues, Lys^428^/Lys^435^, existing in each protein. These residues may interact with amino acid substrates. Lys^146^ accepts acidic amino acids, while Ser^151^ is specifically conjugated to isoleucine.

### 2.4. Gene Ontology Annotations and RNA-Sequence Data Analysis of StGH3 Proteins

**Figure 4:**
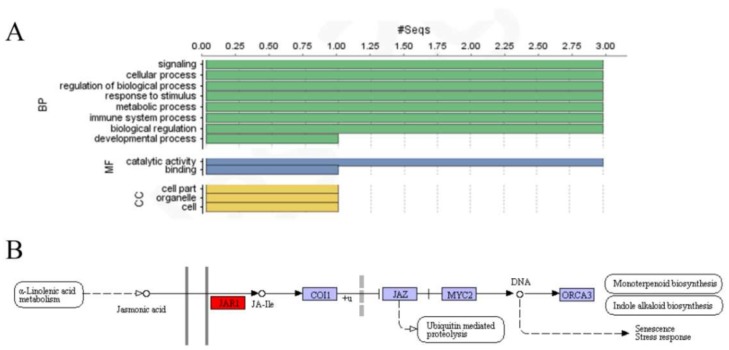
Information from gene ontology annotations and the KEGG pathway. (**A**) GO annotation for StGH3 proteins. BP, MF, and CC indicate Biological Process, Molecular Function, and Cellular Component, respectively. The numbers on the abscissa indicate the number of predicted proteins; and (**B**) the GH3 proteins are related to the KEGG pathway. StGH3.1, StGH3.5, and StGH3.12 are predicted to be part of the JA pathway. The red box is a functional site for of these three proteins according to prediction. COI1 is a receptor [41] which can combine with ASK (*Arabidopsis* Skp-like protein) and AtRbx (*Arabidopsis* ring-box protein) to form the complex SCF^COI1^ [42]. Jasmonate ZIM (JAZ) is a JA pathway suppressor. In the absence of JA, these proteins interact with MYC proteins to block their activity. The bHLH transcription factor MYC is a master regulator of the response to the JA pathway [43]. ORCA3 regulates basic and secondary metabolism of plants by JA induction [44].

To better understand the biological processes affected by the GH3 proteins, we selected potato GH3 proteins for GO analysis using the NCBI database. Three StGH3 proteins (StGH3.1, StGH3.5, and StGH3.12) appeared to participate in multiple signal transduction pathways (signal transduction, response to stress, small molecule metabolic process, and immune system process). Moreover, these three proteins had predicted ligase activity. StGH3.5 appeared to participate in enzyme binding and nucleotidyl transferase activity. The prediction of genes’ cellular component showed that StGH3–5 participate in the component development of the vacuole (Figure 4A). Compared with the databases in the KEGG website, proteins encoded by the *StGH3* group I genes (except *StGH3.11*) participate in the JA pathway (Figure 4B, Table S3). Therefore, these three proteins had important roles in JA adenylation.

**Figure 5:**
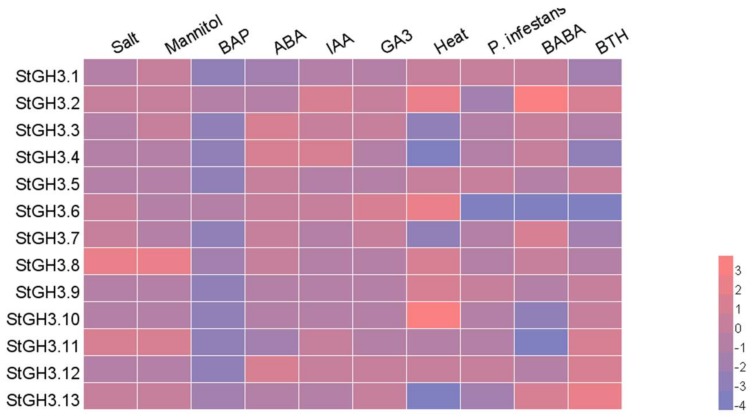
The heatmap based on the RNA-seq database under 10 treatments, which include biotics, abiotics, and hormones. The RNA-seq database was processed by log2 and stress, and hormonal data were compared with the control data. In the heat map, upregulated expression is in **red** and downregulated expression is in **blue**.

The potato expression database was downloaded from the PGSC website which contains transcription sequence expression changes under biotics, abiotics, and hormone treatments [45]. We selected *StGH3* transcriptional sequences to analyze their expression patterns upon various treatments (Table S4 [45]). We processed the RNA-seq database and generated a heatmap (Figure 5). Ten different treatments were part of this analysis. All *StGH3* genes were downregulated upon 6-Benzylaminopurine (BAP) treatment and slightly enhanced or reduced in the presence of the *Phytophthora infestans*. The *StGH3.2* sequence was upregulated by seven stressors, and three treatments increased its expression. The *StGH3.10* sequence was strongly upregulated, and the *StGH3.13* sequence was downregulated to the point of no signal upon heat treatment. Combined GO annotation and RNA-seq data analysis showed that *StGH3.1*, *StGH3.5*, and *StGH3.12* participated in stress regulation. These genes were all upregulated under heat and biotic stresses. The change of StGH3 protein expression regulated by JA has not been reported, and these results should be analyzed following treatment with JA or MeJA.

### 2.5. Expression Analysis of StGH3 Group I Genes

To demonstrate the change of GH3 proteins in plant tissues and under MeJA treatment, qRT-PCR analysis was used to conduct expression analysis. We mainly focused on the group I genes which were related to the JA pathway by prediction. Some researchers used MeJA to treat potato tuber slices and tissue culture seedling. Results showed that JA and MeJA induced dilation of the potato tuber cells and that a single treated stalk of potato could promote tuber formation [46,47]. However, according to Hannapel et al., *StBEL5* is the main gene responsible for regulating potato tuber formation, and JA only has an indirect role in tuber formation [48]. Therefore, we verified that these candidate proteins respond to JA and analyzed their function in different plant tissues by using qRT-PCR.

**Figure 6:**
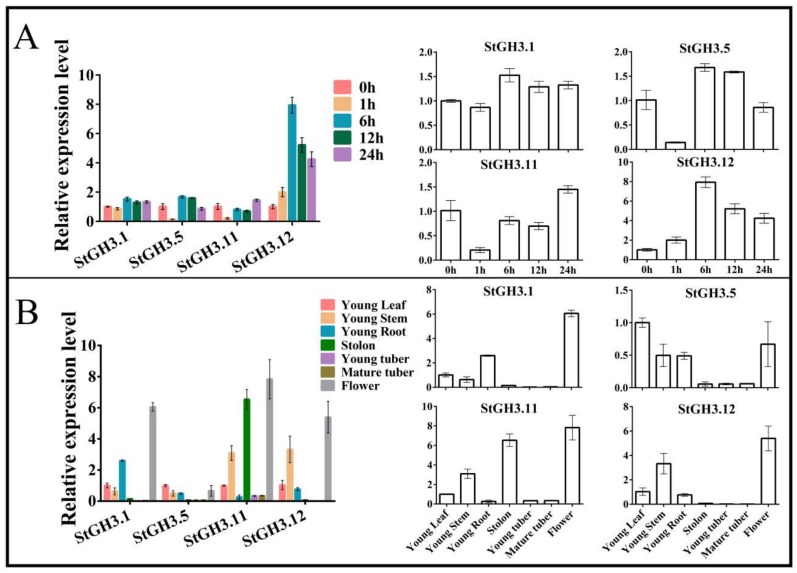
*StGH3* gene expression in different tissues and expression changes upon MeJA treatment for different time periods. (**A**) Expression changes upon MeJA treatment for different time periods; and (**B**) Expression in different tissues.

The tissue culture seedling was treated by 10^−5^ M MeJA [49,50], and the collected materials were treated for different time periods. The expression of the StGH3 group I genes was studied during these treatments (Figure 6A, Table S5). These four genes were all upregulated at different times. Compared with an untreated sample, *StGH3.1*, *StGH3.5*, and *StGH3.12* were upregulated 6 hours after treatment. The expression level gradually decreased from 6 hours to 24 h. The highest change in expression occurred in *StGH3.12*: expression was eight-fold higher than in the control after 6 hours of treatment. Overall, the response time of genes to MeJA treatment was about 6 h. The most responsive gene was *StGH3.12*, and other proteins typically had no obvious response, especially *StGH3.11*, which was downregulated by MeJA treatment. However, if a gene was downregulated in one hour, it might be due to MeJA concentration because the JA compound was poorly soluble in MS liquid medium.

In addition, we studied the expression of these four genes in seven different tissues to verify whether if there was a specific expression in a particular potato tissue (Figure 6B, Table S5). We selected the young leaf as the standard to process the qRT-PCR data by using the 2^−∆∆*C*t^ method. Three genes had greater expression in flowers. Only *StGH3.5* had lower expression in each tissue. The expression level of *StGH3.11* was also high in the stolon and the other genes only had low expression. None of these genes had high expression levels in young tubers or mature tubers. Some researchers showed that JA and MeJA could expand the tuber cells, which leads to potato disks becoming extremely swollen [46,47]. However, the expressions of the StGH3 genes were much lower in the tubers. This may prove that StGH3 proteins from group I could not regulate the tuberization, but they do participate in the JA biological processes of growth regulation.

## 3. Materials and Methods

### 3.1. Mining GH3 Genes from Various Species

Genes, proteins, and transcript sequences from 48 species of algae, mosses, ferns, gymnosperms, and angiosperms were downloaded from the NCBI. Blast 2.6.0 was used to search for homologous sequences based on *AtGH3* [16] as query with *E*-value ≤ 10^−10^. The acquired data were uploaded to the NCBI-CDD website (http://www.ncbi.nlm.nih.gov/cdd) and Pfam website (http://pfam.xfam.org/) to search the domain. All identified GH3 genes were aligned using the multiple sequence alignment tool ClustalX2 (http://www.clustal.org/clustal2/). After excluding small portions of genes with divergent sequences, the others were considered putative genes.

### 3.2. Structure Analysis of GH3 Proteins

To better understand the GH3 proteins, a portion of the structure of GH3 proteins was analyzed. The structure of these proteins was predicted from protein motifs using MEME (http://meme-suite.org/tools/meme), with the parameters set to default, only selecting the number of motifs (20). Predictions of proteins’ secondary structure came from PHDsec server, which could upload the data onto PRABI Lyon Gerland website (https://prabi.ibcp.fr/htm/site/web/home), and DNAMAN software (https://www.lynnon.com/pc/framepc.html) was used to analyze multiple sequence alignment.

### 3.3. Construction of the Phylogenetic Tree

Phylogenetic analysis of the GH3 family was conducted using MEGA7 software (https://www.megasoftware.net/). Seventeen species of plants were included in the phylogenetic tree. Phylogenetic trees were produced using the Neighbor-Joining (NJ) method [39] with the following parameters: 1000 bootstrap replications, Poisson model, and pairwise deletions.

### 3.4. Analysis of Gene Duplications

Tandemly duplicated gene pairs were identified by comparing their physical locations on chromosomes and their homology (more than 50%). We defined paralogous genes as those existing in the same chromosome within a 50 kb physical distance, indicating tandem duplicated pairs [40,51]. The segmental duplication of each gene was ensured through the Plant Genome Duplication Database (PGDD) website (http://chibba.agtec.uga.edu/duplication/). Ka/Ks values were calculated using DnaSP6 software [52].

### 3.5. GO Annotation and RNA-Seq Data Analysis

Blast2GO software (https://www.blast2go.com/) was used to analyze the gene ontology (GO). The full-length amino acid sequences were uploaded into the program, and the NCBI database was chosen as the reference to analyze the molecular function, cellular components, and biological processes. The KEGG pathway of proteins could be searched on the KEGG website (http://www.kegg.jp/kegg/kegg2.html).

The RNA-seq data [53] were downloaded from the PGSC website (http://solanaceae.plantbiology.msu.edu/pgsc_download.shtml). The selected raw data were transformed by log2, and then HemI software [54] was used to visualize the expression.

### 3.6. Plant Growth Conditions and Treatments

The potato material (DM1-3-516-R44) was grown with 20 g/L sugar on solid MS medium with vitamins (PhytoTech, Shawnee Mission, MS, USA) as a culture medium for four weeks in a plant incubator at 25 ± 1 °C under 10,000 lx in light for 16 h and 20 ± 1 °C under 0 lx for 8 h. For the MeJA treatment, 10^−4^ M MeJA was added to liquid MS medium with 20 g/L sugar. After culturing for 1 h, 6 h, 12 h, and 24 h, the samples were taken to analyze *GH3* gene expression.

B5141-6 (Lenape) × Wauseon was planted in the greenhouse for 3 months. Seven tissues (young leaf, young stem, young root, stolon, young tuber, mature tuber, and flower) were selected to test gene expression.

### 3.7. Expression Analysis of the StGH3 Genes

Plant total RNA (Invitrogen, Carlsbad, California, USA) from four plants treated for different times with MeJA and RNA from seven different tissues were used for reverse transcription into cDNA (Takara, Tokyo, Japan). Elongation factor 1-a (*ef1-a*) was used as a reference gene to quantify the expression of the *StGH3* genes [55]. Bio-Rad Real-Quantitative real-time PCR analysis System (CFX96, Hercules, CA, USA) was used to analyze the expression levels. Gene expression analysis upon each treatment was based on three technical replicates. The relative expression levels were calculated using 2^−∆∆*C*t^ [56].

## 4. Conclusions

We performed an evolutionary analysis of the GH3 protein family in the plant kingdom to reveal gene structure, phylogenetic relationships, and the evolution of GH3 proteins in each group. Group I and II genes were found in mosses. Group I contains many ancient genes. Therefore, we conclude that their function was indispensable to all plant species. Multiple group II genes appeared in seed plants. Therefore, we could infer that group II genes play crucial roles in plants. Group III members did not appear until the angiosperm period. We predict that group III is the most recent group and is closely related to group II. This conclusion will provide a reference for the evolutionary relationships of GH3 proteins in plants. Our analysis also revealed that group I genes were related to the JA response, and several genes were also involved in physiological processes of various tissues and responded to some types of stress in the potato. The results of this study increase our understanding of the evolutionary relationships in the GH3 family and also serve as the basis for the functional identification of potato GH3 proteins. In subsequent studies, we will try to reveal the function of GH3 proteins in the JA biological process and improve the response to some stress types through JA regulation in the potato.

## Figures and Tables

**Table 1:** Glycoside Hydrolase 3 (*GH3*) genes identified in 48 sequenced plant genomes.

Lineage		Organism	Genome Total Length (Mb) ^1^	Numbers of GH3 Protein	Numbers of Group I ^2^	Numbers of Group II ^3^	Numbers of Group III	Tandem Duplication (Pairs) ^4^	Segmental Duplication (Pairs) ^5^	References
Algae		*Chlamydomonas reinhardtii*	120.405	0	0	0	0	0	0	This study
		*Volvox carteri*	137.684	0	0	0	0	0	0	This study
Mosses										
		*Marchantia polymorpha* L.	205.718	2	1	1	0	0	0	Bowman et al. [10]
		*Physcomitrella patens*	477.948	2	2	0	0	0	0	Nicole et al. [9]
Ferns										
		*Selaginella moellendorffii*	212.502	18	9	9	0	2	1	This study
Gymnosperms										
		*Picea abies*	28354	7	1	6	0	0	0	This study
Angiosperms										
	*Amborellaceae*	*Amborella trichopoda* ^#^	706.495	6	2	4	0	0	0	This study
Eudicots										
	*Chenopodiaceae*	*Beta vulgaris*	482.053	5	1	4	0	0	1	This study
	*Brassicaceae*	*Arabidopsis lyrata*	204.898	17	2	8	7	10	0	This study
		*Arabidopsis thaliana*	116.846	19	2	8	9	11	2	Jain et al. [4]
		*Brassica oleracea*	501.692	35	4	11	20	10	13	This study
		*Brassica rapa*	284.129	38	5	11	22	8	13	This study
		*Capsella rubella*	133.064	20	2	8	10	12	3	This study
	*Solanaceae*	*Capsicum annuum*	2935.88	11	4	7	0	0	1	This study
		*Solanum lycopersicum*	760.067	17	11	6	0	6	4	This study
		*Solanum tuberosum*	705.934	13	4	9	0	3	4	This study
	*Leguminosae* sp.	*Cajanus cajan*	620.626	12	4	8	0	0	0	This study
		*Cicer arietinum*	520.885	7	2	5	0	0	1	This study
		*Glycine max*	953.339	24	8	16	0	0	36	This study
		*Lotus japonicus*	394.455	7	1	6	0	0	0	This study
		*Medicago truncatula*	412.924	17 *	6	7	0	7	2	Yang et al. [6]
		*Phaseolus vulgaris*	535.413	12	4	8	0	0	7	This study
	*Rosids*	*Fragaria vesca*	214.373	8	2	6	0	0	0	This study
		*Malus domestica*	1288.87	15	5	10	0	1	4	Yuan et al. [5]
		*Prunus mume*	234.03	7	2	5	0	0	1	This study
		*Prunus persica*	212.767	7	2	5	0	0	1	This study
		*Pyrus bretschneideri*	508.551	15	2	13	0	0	4	This study
	*Cucurbitaceae*	*Citrullus lanatus*	321.047	8	2	6	0	1	0	This study
		*Cucumis sativus*	323.986	10	3	7	0	1	0	This study
	*Actinidiaceae*	*Actinidia chinensis*	604.217	15	4	11	0	0	3	This study
	*Caricaceae*	*Carica papaya*	370.419	6	2	4	0	0	0	This study
	*Euphorbiaceae*	*Ricinus communis*	350.622	7	2	5	0	0	0	This study
	*Lentibulariaceae*	*Utricularia gibba*	100.689	6	2	4	0	0	1	This study
	*Malvaceae*	*Theobroma cacao*	335.437	19	2	17	0	8	3	This study
		*Gossypium raimondii*	767.667	32	6	10	16	12	11	This study
		*Eucalyptus grandis*	691.43	9	3	6	0	0	2	This study
	*Nelumbonaceae*	*Nelumbo nucifera*	797.494	11	4	7	0	0	4	This study
	*Rutaceae*	*Citrus sinensis*	323.528	9	2	7	0	1	0	This study
	*Salicaceae*	*Populus trichocarpa*	417.287	13	4	9	0	0	8	This study
	*Vitaceae*	*Vitis vinifera*	486.197	9	3	6	0	0	1	Bottcher et al. [35]
Monocots										
	*Poaceae*	*Brachypodium distachyon*	218.345	9	2	6	1	0	1	This study
		*Hordeum vulgare*	1825.17	5	1	4	0	0	0	This study
		*Oryza sativa*	374.423	13	4	9	0	0	1	Jain et al. [4]
		*Triticum urartu*	3747.05	9	3	6	0	0	0	This study
		*Zea mays*	2145.45	13	5	8	0	1	3	Feng et al. [36]
	*Arecaceae*	*Elaeis guineensis*	1017.1	16	5	11	0	0	2	This study
	*Musaceae*	*Musa acuminata*	472.231	18	8	10	0	1	18	This study
	*Orchidaceae*	*Phalaenopsis equestris*	1064.2	6	3	3	0	0	0	This study
Total				579	159	339	85			

^1^ The data are from NCBI (www.ncbi.nlm.nih.gov/genome/); ^2^ Group I represents proteins that may use jasmonic acid (JA) as a substrate to participate in the JA biological pathway; ^3^ Group II proteins may participate in the regulation of indolacetic acid (IAA); ^4^ JGI (https://phytozome.jgi.doe.gov/pz/portal.html) contains hole information; ^5^ Related information obtained from PGDD (http://chibba.agtec.uga.edu/duplication/); ^#^ *Amborella trichopoda* was known as the sister group of the remaining flowering plants in molecular phylogenetic analyses [37]; * Yang et al. showed that GH3 proteins were four separated subfamilies in *Medicago truncatula*, but *MtGH3.14* was missing [6]. Subfamilies 1 and 3 could be considered as the same family according to the family classification of *Arabidopsis* [4]. Therefore, six proteins belonged to group I, seven proteins belonged to group II, and no proteins belonged to group III. The remaining three proteins were grouped separately.

## Supplementary Materials

Supplementary materials can be found at http://www.mdpi.com/1422-0067/19/7/1850/s1.
